# Towards a Structural Comprehension of Bacterial Type VI Secretion Systems: Characterization of the TssJ-TssM Complex of an *Escherichia coli* Pathovar

**DOI:** 10.1371/journal.ppat.1002386

**Published:** 2011-11-10

**Authors:** Catarina Felisberto-Rodrigues, Eric Durand, Marie-Stéphanie Aschtgen, Stéphanie Blangy, Miguel Ortiz-Lombardia, Badreddine Douzi, Christian Cambillau, Eric Cascales

**Affiliations:** 1 Aix-Marseille Université, Architecture et Fonction des Macromolécules Biologiques, Campus de Luminy, Marseille, France; 2 CNRS, Architecture et Fonction des Macromolécules Biologiques, UMR 6098, Campus de Luminy, Marseille, France; 3 Laboratoire d'Ingénierie des Systèmes Macromoléculaires, Institut de Microbiologie de la Méditerranée, CNRS UPR9027, Aix-Marseille Université, Marseille, France; Yale University School of Medicine, United States of America

## Abstract

Type VI secretion systems (T6SS) are trans-envelope machines dedicated to the secretion of virulence factors into eukaryotic or prokaryotic cells, therefore required for pathogenesis and/or for competition towards neighboring bacteria. The T6SS apparatus resembles the injection device of bacteriophage T4, and is anchored to the cell envelope through a membrane complex. This membrane complex is composed of the TssL, TssM and TagL inner membrane anchored proteins and of the TssJ outer membrane lipoprotein. Here, we report the crystal structure of the enteroaggregative *Escherichia coli* Sci1 TssJ lipoprotein, a two four-stranded β-sheets protein that exhibits a transthyretin fold with an additional α-helical domain and a protruding loop. We showed that TssJ contacts TssM through this loop since a loop depleted mutant failed to interact with TssM *in vitro* or *in vivo*. Biophysical analysis of TssM and TssJ-TssM interaction suggest a structural model of the membrane-anchored outer shell of T6SS. Collectively, our results provide an improved understanding of T6SS assembly and encourage structure-aided drug design of novel antimicrobials targeting T6SS.

## Author Summary

Type VI secretion systems (T6SS) are specialized secretion machines responsible for the transport of virulence factors. T6SS are versatile as they are able to target both eukaryotic and prokaryotic cells. They therefore play an important role in pathogenesis by acting directly on the host, as well as eliminating competing bacteria from the niche. At a molecular level, T6SS are composed of a minimum of 13 proteins called core-components, all required for the activity of the secretion system. These core-components can be divided in two groups: soluble proteins having a common evolution history with bacteriophage T4 subunits, and membrane or membrane-associated proteins required for anchoring the bacteriophage-like structure to the envelope. Here, we report the crystal structure of one of the membrane-associated core component, the TssJ lipoprotein. The structure exhibits a transthyretin fold supplemented with additional structural elements. One of these, a loop connecting two beta-strands, is responsible for the interaction of the TssJ lipoprotein with the C-terminal domain of the inner membrane protein TssM. We propose that these two proteins link the two membranes and form a channel accommodating the bacteriophage-like structure. These results provide important new insights to understand the biogenesis of these secretion apparati.

## Introduction

Pathogenic bacteria have evolved numerous and original strategies to invade host tissues, colonize new niches or to kill predators. Bacteria are able to adhere to various surfaces and to actively release protein toxins. The delivery of effectors in the milieu, into host cells or bacteria involves dedicated machineries called secretion systems. Among the six secretion systems identified in Gram negative bacteria, the recently identified Type VI secretion system (T6SS) is composed of 13 core components which form a trans-envelope apparatus [1]. The T6SS are highly versatile in terms of functions [1]–[4]. T6SS have been found to be required for resisting predation or for pathogenesis in several bacteria: in *Vibrio cholerae*, the T6SS is required to escape amoeba predation, or for killing host cells by modification of the host cell cytoskeleton and subsequent impairing phagocytic activity [5]–[6]. Beside the role of several T6SS in pathogenesis towards animal or plant models, it was recently reported that T6SS are involved in stress sensing, in regulating bacteria-bacteria interactions or in targeting other bacterial cells, and may therefore help in competition towards a specific niche [2], [7], [8]. When not required for pathogenesis, T6SS yet provide a critical advantage to neighbouring bacteria, allowing an improved colonization efficiency.

A hallmark of T6SS is that two proteins are found in culture supernatants of bacteria producing T6SS: Hcp and VgrG [1]. The crystal structures of Hcp and VgrG have been reported: Hcp forms hexameric rings leaving a pore of ∼40 Å [9] whereas three VgrG assemble to form a syringe-like structure [10]–[12]. Phylogenetic and structural data have shown that these two proteins share remarkable homologies with bacteriophage components. The Hcp structure is superimposable to the major tail protein gpV of bacteriophage λ (bacteriophage T4 gp19 protein; [9], [13]) whereas VgrG has a fold highly similar to the gp27-gp5 complex, the cell puncturing device of bacteriophage T4 [10], [11], [14]. Several other subunits of Type VI secretion systems also share a common evolutionary history with other bacteriophage baseplate or sheath components [1], [15]. These include TssE, a homologue of the baseplate gp25 protein, and TssB and TssC, which have been shown to form tubular structures resembling the bacteriophage tail sheath (the nomenclature used in this manuscript follows the general Tss nomenclature [16]). Interestingly, the *Vibrio cholerae* TssB/TssC (VipA/VipB) tubular structures are disassembled by TssH, an AAA+ traffic ATPase of the Clp family [17]. The current model suggests that these proteins may assemble an extracellular tubular structure composed of the Hcp protein carrying the VgrG protein at the tip [18]. This upside-down bacteriophage structure will thus deliver the VgrG protein in the milieu or into host cells [12], [19]. Several VgrG proteins carry an additional C-terminal domain which acts as an effector module with functions interfering with the host cytoskeleton or the host physiology [12].

Beside bacteriophage-derived components, a number of membrane-associated proteins were shown to be critical for T6SS. Among these components, TssL and TssM have close homologues in Type IVb secretion systems [1], [15], [20]. Two other T6SS genes, *tssJ* and *tssH* encode an outer membrane (OM) lipoprotein and an AAA+ ATPase, two components regularly found in bacterial secretion systems or in trans-envelope structures allowing the assembly of cell surface appendages [1], [21]. An immunoprecipitable complex composed of four proteins, TssJ, TssL, TssM and TagL has been evidenced in enteroaggregative *Escherichia coli* (EAEC) [22], [23]. TssM is an inner membrane (IM) protein with three transmembrane segments. Homologues of TssM in *Agrobacterium tumefaciens* and *Edwardsiella tarda* have been shown to interact with homologues of the TssL inner membrane protein and of the outer membrane lipoprotein TssJ [20], [24]. TssL interacts with TagL, an inner membrane protein carrying a peptidoglycan-binding motif of the OmpA/Pal/MotB family that anchors the T6SS to the cell wall [22], [23]. This membrane complex therefore links both membranes and the peptidoglycan layer. Although *in vivo* data have been accumulated on the topology of the membrane complex subunits and their interactions, little is known on the structural organization of these proteins. To gain structural information on the assembly of this complex, we initiated the purification of the different subunits. We report here the crystal structure of the TssJ protein of the enteroaggregative *Escherichia coli* Sci1 T6SS which likely constitute a prototype for all TssJ-like proteins. We also present biochemical data on the TssM protein and on the TssJ-TssM interaction. We provide *in vitro* and *in vivo* evidence for the function of a specific loop of TssJ in mediating contact with the TssM subunit, which therefore provide fundamental insight into T6SS biogenesis and topology.

## Results

### Crystal structure of the EAEC TssJ protein

A fragment of the *tssJ* gene of the *sci1* cluster from enteroaggregative *Escherichia coli* consisting of amino-acid residues 2-155 of the processed TssJ lipoprotein (residues 25-178 of the full-length protein) was cloned in the Gateway vector pETG20A, with an N-terminal fusion hexahistidine tagged thioredoxin for purification [25]. This construct consists of a polypeptide chain starting at the glycine residue following the cysteine anchoring TssJ to an acyl chain. The numbering used in this report follows the sequence of the mature lipoprotein, between residues Cys1 (here mutated in Gly) and Lys155. The TssJ protein was purified by affinity chromatography and gel filtration, and the native TssJ protein was obtained upon fusion and tag cleavage by the TEV protease.

TssJ was analyzed by MALS/QELS/UV/RI (on-line multi-angle laser light scattering/quasi-elastic light scattering/absorbance/refractive index detectors) experiments [26]. The protein was shown to be a monomer at a concentration of 4 mg/mL (230 µM) at pH 7.5 in the presence of 100 mM NaCl. Mass and hydrodynamic radius calculation performed with the ASTRA software (Wyatt Technology) using a *dn/dc* value of 0.185 mL/g indicated a mass of 17260±800 Da, close to the theoretical mass of 16,899 Da (Figure S1, Tables S1 & S2).

TssJ crystallized readily with 2.2 M ammonium sulfate as a precipitant at pH 6.0 in sitting nano-drops [27]. We collected a native dataset and a dataset from a crystal soaked in CsI/NaI at beamline Proxima 1 (Soleil synchrotron, Saint-Aubin, France). The structure was solved from 2.0 Å resolution SIRAS (single isomorphous replacement with anomalous scattering) maps calculated using CsI as phasing agents, and the resolution limit was extended to 1.35 Å with the native data set (Figure S2, Table S3). The polypeptide chain could be traced from residue Ile23 to Pro151. The segment 1-22 anchoring the protein to the membrane *via* Cys1 and its phospholipid thioester as well as the last four residues were not ordered in the crystal.

A unique TssJ molecule is contained in the asymmetric unit, and the PISA server [28] did not identify any sufficient interactions between TssJ molecules related by crystallographic symmetry. We can therefore conclude that TssJ is a monomer in solution.

**Figure 1:**
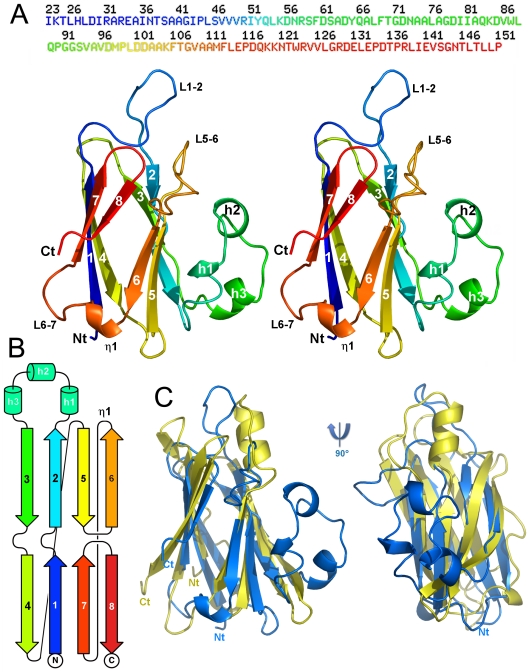
Structure of the enteroaggregative *E. coli* T6SS TssJ subunit. (**A**) Stereoview of TssJ in ribbon representation and rainbow coloring, from blue (N-term) to red (C-term); the sequence is represented above. Figure made with Pymol [61]. (**B**) Topology cartoon of TssJ (same coloring as in (A)). (**C**) Structural comparison of TssJ and its nearest homologue, transthyretin (1sn5), after superimposition. The topology is identical for both proteins and their β-sandwiches superimpose within 3.2 Å. Note the presence of an extra helical domain in TssJ, and an extra helix (top) in transthyretin.

TssJ has the topology of a β-sandwich formed by two four-stranded β-sheets (Figure 1). Sheet one is composed or β-strands 4(-1), 1, 7 and 8 (1), and is packed against sheet 2 which contains β-strands 3(-1), 2, 5(-1) and 6. The other face of β-sheet 2 one is covered in part by three short helices (h1-3) occurring between β-strands 2 and 3. These helices exhibit B-factors larger than average, in particular the segment 56-71, between helices 2 and 3 which has very weak electron density. Of particular interest are the loops located between strands 1 and 2, and between strands 5 and 6. Another long loop incorporates the η1 helix between strands 6 and 7.

Searching the protein database for structurally related proteins with Dali [29] returned significant hits with transthyretin (1sn5 [30]; Z = 6.4; rmsd [root mean-square deviation] = 3.2 Å) on 87 residues among 116 involved in comparison (Figure 1C) and with 5-hydroxy-isourate hydrolase (3iwu [31]; Z = 6.3; rmsd = 3.3 Å). Both proteins originate from vertebrates, a mammalian blood transport protein and an enzyme from zebra fish, respectively [30], [31]. Dali also returned the recently determined structure of the ExsB lipoprotein from the *P. aeruginosa* Type III secretion system (T3SS) [32], although with lower scores (2yjl; Z = 5.2; rmsd = 4.0 Å; Figure S3). Noteworthy, TssJ helical domain (residues 57-71) is absent in transthyretin, 5-hydroxy-isourate hydrolase and ExsB, but a single helix is located on the same face, between strands 4 and 5 of transthyretin (Figure 1C). The extended loop (residues 38-45) between stands 1 and 2, forming a protruding extension, is also absent in the three proteins (Figure 1C, Figure S3). In the crystal, this loop points out of the core of the protein and is stabilized by contacts with a symmetry related molecule, although this contact is not biologically relevant due to its limited interface.

Sequence alignments of TssJ from the enteroaggregative *E. coli* Sci1 have been performed with the 49 closest sequences in the non redundant (NR) database (Figure S4). The 33 first sequences do not present insertions nor deletions, while the 17 more divergent ones exhibit a 3-residue insertion in the loop between stands 1 and 2, making this loop even longer. On the 16 conserved residues, 13 are present in the X-ray structure (Figure S5). The two first residues (Cys1 and Gly2) are of functional importance in TssJ proteins: Cys1 is the N-terminal acylated residue whereas Gly2 is responsible for the Lol-dependent outer membrane targeting [21]. Three conserved prolines (at positions 90, 99 and 116) are involved in structural integrity of strands-joining loops. A group of aromatic residues, Tyr65, Phe113 and Trp123 form also a structurally important hydrophobic cluster stabilizing the α-helical domain against the β-sandwich core. The other residues are scattered along the polypeptide chain and do not reveal interpretable features.

Analysis using the CASTp software [33] did not identify significant surface cavities with a volume larger than 100 Å^3^. In contrast, when the TssJ structure is overlaid with transthyretin and ExsB (Figure 1C, Figure S3), two domains protrude from the core of the protein: the loop between strands 1 and 2 (residues 38-45) and the helical domain (residues 57-81), the latter one presenting very high B-factors, especially between residues 65 and 75 (Figure S6A). The calculation of contact electrostatic potential did not highlight any hydrophobic or positively or negatively charged surface patches, but indicated a scattered and balanced charges distribution over the whole surface (Figure S6B).

### TssM ekto-domain and sub-domains production and characterization

Enteroaggregative *E. coli* TssM is a large - 1129 amino-acids - protein with an N-terminal cytoplasmic domain (1-387) bearing three trans-membrane helices and a periplasmic domain of 744 residues (termed hereafter ekto-domain; Aschtgen and Cascales, unpublished data). JPRED [34] secondary structure predictions reveal that the first ∼500 residues (386-930) of the ekto-domain are helical while the C-terminus (931-1129) is essentially a β-domain (Table S2). We expressed three constructs of TssM domains in fusion with Trx and His tags: the ekto-N-terminal domain (ekto-Nt, 386-930), the ekto-C-terminal domain (ekto-Ct, 931-1129) and the full-length ekto domain (386-1129). The ekto-Nt domain was well expressed and soluble up to 0.7 mg/mL without the need of detergent addition. The ekto-Ct domain was produced as inclusion bodies and could not be purified. The full-length TssM-ekto was expressed in large quantities and remained soluble after TEV cleavage. It could be concentrated up to 9.0 mg/ml without the need of detergent. The CD spectra of these domains indicated that TssM-ekto-Nt is predominantly formed of α-helices, while a contribution of β-strands appears in the full-length TssM (Table S2, Figure S7A).

### *In vivo* and *in vitro* interaction of TssJ with TssM

**Figure 2:**
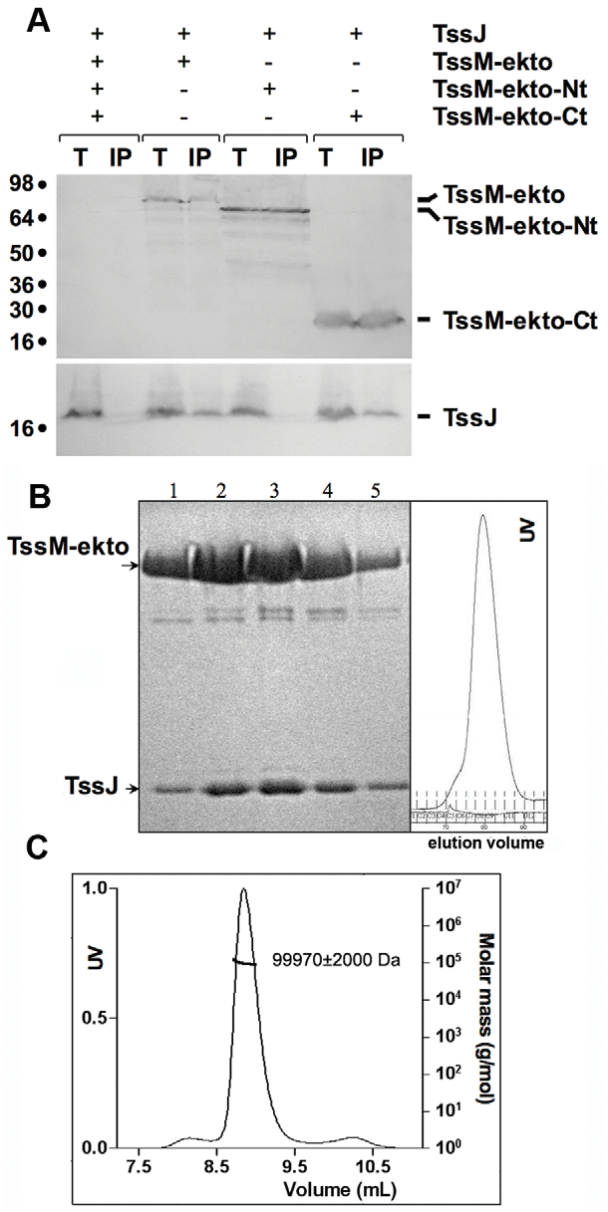
TssJ interacts with the C-terminal domain of TssM. (**A**) Solubilized extracts of *E. coli* K12 W3110 strain producing (+) or not (-) HA-tagged TssJ and FLAG-tagged TssM-ekto or -Nt or –Ct derivatives were subjected to immunoprecipitation with anti-FLAG-coupled beads. The total solubilized material (T) and the immunoprecipitated material (IP) were loaded on a 12.5%-acrylamide SDS PAGE, and immunodetected with anti-HA (TssJ; lower panel) and anti-FLAG (TssM-ekto and sub-domains; upper panel) monoclonal antibodies. Immunodetected proteins are indicated on the right. Molecular weight markers are indicated on the left. (**B**) Gel filtration showing the direct interaction of TssM-ekto with TssJ. The SDS-PAGE analysis of the fractions is shown on the left panel. The chromatogram of the gel filtration is shown on the right panel. (**C**) MALS/QELS/UV/RI analysis of the TssM-ekto/TssJ complex.

Since the TssJ lipoprotein has been proposed to facing the periplasm [21], we tested whether TssJ interacted with TssM-ekto in an *in vivo* co-immunoprecipitation assay. TssM-ekto and the two deletion variants, TssM-ekto-Nt and TssM-ekto-Ct were cloned downstream a signal peptide allowing their targeting to the periplasm. All TssM constructs were fused to a FLAG epitope. The full-length, acylated TssJ protein was produced with a C-terminal hemagglutinine (HA) tag [21]. Both proteins were produced from compatible plasmids in *E. coli* K12 (*i.e.*, devoid of T6SS gene cluster) to test for direct interaction. Cells expressing both TssM-ekto and TssJ proteins were treated with formaldehyde and subjected to membrane solubilization. Solubilized extracts were then used in an immunoprecipitation assay with the anti-FLAG antibody. TssJ co-precipitated with TssM, whereas no TssJ was found associated with the resin in absence of TssM-ekto (Figure 2A). These results demonstrate that TssJ interacts with the periplasmic domain of TssM *in vivo*. Similar experiments performed with the TssM-ekto sub-domains further showed that TssJ interacts with TssM-ekto-Ct but not with the TssM-ekto-Nt variant (Figure 2A).

Purified TssJ and TssM-ekto produced in *E. coli* were tested in an *in vitro* interaction assay. Both proteins were mixed with a slight molar excess of TssJ and were subjected to gel-filtration. A peak was observed at an elution time slightly shorter compared to that of TssM-ekto alone, and analysis of this peak by SDS page revealed the presence of both partners (Figure 2B). Mass and hydrodynamic radius calculations confirmed the formation of the TssJ-TssM complex with a mass of 99,970±2,000 Da and a 1∶1 stoichiometry (Figure 2C, Table S2). Together, these results demonstrated that TssM-ekto and TssJ interact directly and form a complex of 1∶1 stoichiometry.

**Figure 3:**
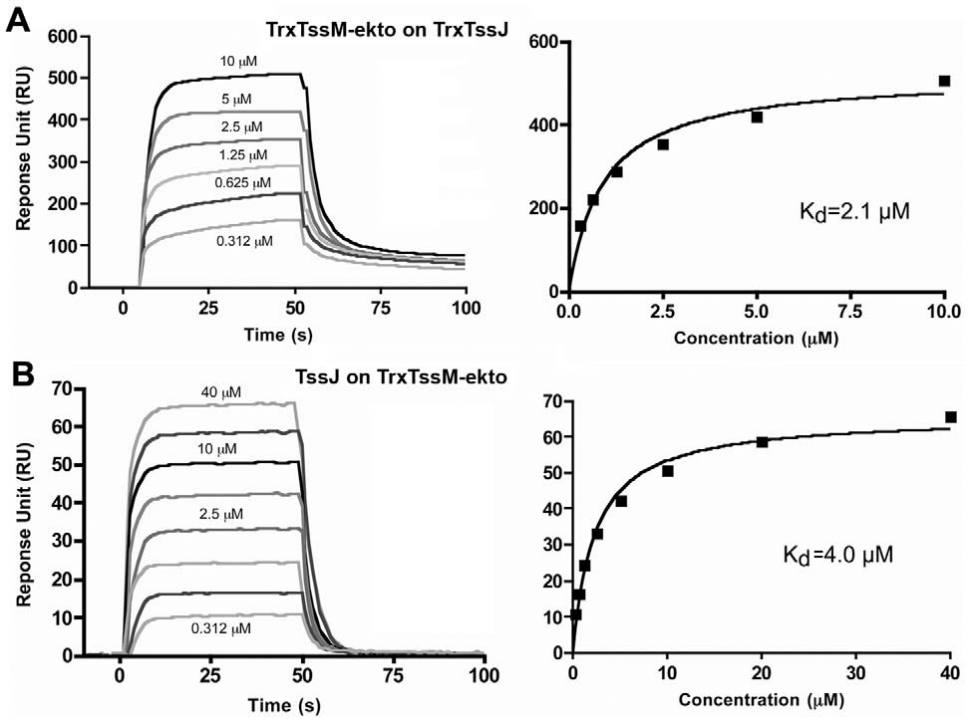
Measure of the interaction between TssM-ekto and TssJ by Surface Plasmon Resonance. (**A**) Sensorgram and saturation curve of the titration of Trx-TssJ by Trx-TssM-ekto. The CM5 chip (BIAcore) was coated with TssJ N-terminal thioredoxine fusion with 600 response units (RU) and the Trx-TssM-ekto was injected in the microfluidic channel. (**B**) Sensorgram and saturation curve of the titration of Trx-TssM-ekto by TssJ. The CM5 chip was coated with TssM-ekto N-terminal thioredoxine fusion with 3000 response units, and TssJ was injected in the microfluidic channel. The K_D_ values were obtained using the fitting tool of the BIAevaluation software (BIAcore).

We analyzed the strength of the interaction by performing a surface plasmon resonance study (SPR) with a Biacore X100. TssJ was covalently coupled to a CM5 chip and TssM-ekto was injected in the microfluidic channel. The sensorgrams indicated that TssM-ekto was released in a short time without the necessity of chip regeneration (Figure 3A). The K_d_ could be calculated from the levels of the sensorgrams at equilibrium, since the values of K_on_ and K_off_ could not be obtained in such a context. The average of three experiments yielded a K_d_ value of 2.1±0.25 µM (Figure 3B). We then performed the symmetrical experiment using covalently linked TssM-ekto to the chip. Upon TssJ injection, a K_d_ value of 4.0±0.5 µM was measured (Figure 3B). In contrast, injection of TssM-ekto-Nt domain on the TssJ-coupled CM5 chip did not result in any signal on the sensorgram, revealing that TssJ does not interact with the TssM N-terminal, α-helical domain *in vitro*. The TssM-ekto-Ct domain was not tested in this assay as it was produced as inclusion bodies and could not be purified. Collectively, the data from the *in vivo* and *in vitro* experiments demonstrate the existence of a 1∶1 stoichiometry complex between the C-terminal periplasmic domain of TssM and the TssJ lipoprotein, with a K_d_ of 2-4 µM.

### TssJ interaction mutants

Interactions between proteins require surface complementarities. The three dimensional structure of TssJ shows that it lacks any crevice able to host any putative TssM-ekto protruding domain. In contrast, TssJ exhibits extensions, compared to transthyretin and ExsB, which could interact with crevices on TssM. The helical domain is located on the face of the β-sandwich opposite to the N- and C-termini, while the extended loop is located at the apical side of the β-sandwich, opposite to the N-terminus, and hence to the outer membrane. These sites are however close enough to participate to a large interaction area with TssM. We therefore constructed TssJ mutants deleted of residues 39-42 from the L1-2 loop (TssJ-ΔL1-2) and of the helical domain (TssJ-Δαdom). The TssJ-ΔL1-2 mutant was expressed at similar level that the native protein, both *in vivo* and *in vitro*. The TssJ-ΔL1-2 mutant was found to be soluble, monomeric, and with a CD spectrum closely similar to that of the native protein (Figure S7B), indicating that the TssJ-ΔL1-2 mutant was properly folded. By contrast, TssJ mutants deleted or partly deleted of the helical domain were little produced *in vivo*, and not produced in *vitro*, precluding further studies with them. An hydrophobic patch at the interface between TssJ core and the helical domain (Phe113, Trp123) might have been exposed to solvent, probably inducing aggregation.

**Figure 4:**
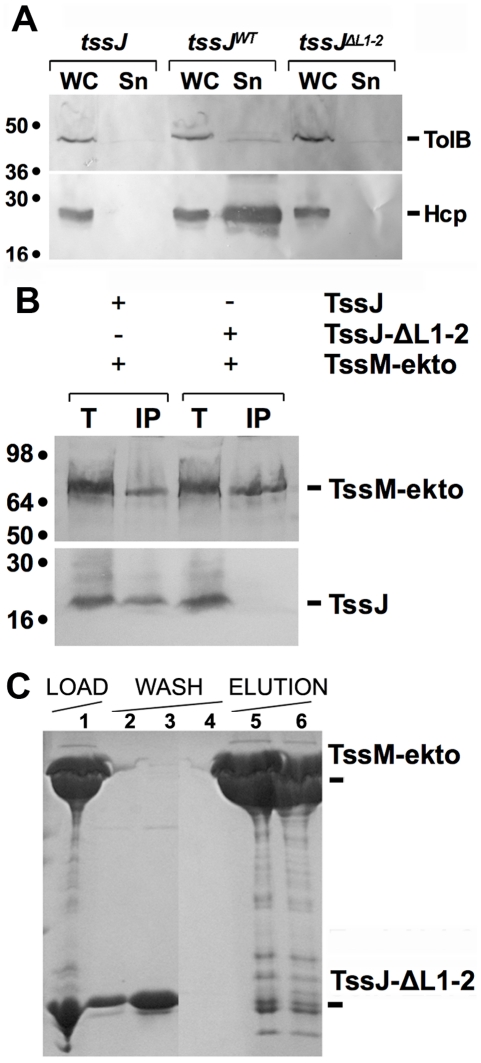
The L1-2 loop of TssJ is required for TssJ-TssM complex formation. (**A**) *In vivo* Hcp release assay. Hcp_FLAG_ release was assessed by separating whole cells (WC) and supernatant (Sn) fractions from *tssJ* cells carrying the empty vector (*tssJ*), the vector encoding wild-type TssJ (*tssJ^WT^*) or the vector encoding the TssJ-ΔL1-2 mutant (*tssJ^ΔL1-2^*). 2 ×10^8^ cells and the TCA-precipitated material of the supernatant from 5×10^8^ cells were loaded on a 12.5%-acrylamide SDS-PAGE and immunodetected using the anti-FLAG monoclonal antibody (lower panel) and the anti-TolB polyclonal antibodies (lysis control; upper panel). (**B**) Solubilized extracts of *E. coli* K12 W3110 strain producing (+) or not (-) FLAG-tagged TssM-ekto and HA-tagged TssJ or TssJ-ΔL1-2 mutant were subjected to immunoprecipitation with anti-FLAG-coupled beads. The total solubilized material (T) and the immunoprecipitated material (IP) were loaded on a 12.5%-acrylamide SDS PAGE, and immunodetected with anti-HA (TssJ and TssJ-ΔL1-2; lower panel) and anti-FLAG (TssM-ekto; upper panel) monoclonal antibodies. Immunodetected proteins are indicated on the right. Molecular weight markers are indicated on the left. (**C**) Affinity purification of TssJ-ΔL1-2 with TRX-His6-TssM-ekto. The Coomassie blue-stained SDS-PAGE shows the fractions of the purification steps (Load, fraction 1; Wash, fractions 2-4; Elution, fractions 5 and 6). The positions of the proteins of interest are indicated on the right.

The TssJ-ΔL1-2 mutant was tested (i) for function in a complementation assay and (ii) *in vivo* and *in vitro* interaction with TssM-ekto. The presence of Hcp in the culture supernatant has been previously shown to reflect the correct assembly of the machine. Whole cells and supernatants of Δ*tssJ* cells expressing full-length wild-type or mutant TssJ were separated by centrifugation and the presence of Hcp in both fractions was assessed by western-blot. As shown in Figure 4A, the wild-type TssJ protein was functional while TssJ-ΔL1-2 did not complement the *tssJ* mutation for Hcp release. We then tested whether the deletion mutant of TssJ interacts with the periplasmic domain of TssM in an *in vivo* co-immunoprecipitation assay. Figure 4B shows that TssJ-ΔL1-2 did not co-immunoprecipitate with TssM-ekto.

This conclusion was validated by *in vitro* analyses. First, analysis of a mixture of TssM-ekto and TssJ-ΔL1-2 by gel filtration revealed a unique peak eluting at the same volume as TssM-ekto alone. SDS-PAGE analysis of the different fractions demonstrated the presence of TssM only (Figure 4C). We then performed SPR experiments with TssM-ekto covalently linked to a CM5 chip. Whereas we detected a 2-4 µM interaction between TssM-ekto and TssJ, injection of the TssJ-ΔL1-2 mutant at a concentration up to 10 µM failed to produce any deviation of the sensorgram, indicating thus a lack of interaction between TssM and TssJ-ΔL1-2. Coupled to the observation that TssJ-ΔL1-2 was correctly folded, we concluded that the L1-2 loop of TssJ is a critical determinant for the TssJ-TssM interaction.

## Discussion

Type VI secretion systems are important determinants of bacterial pathogenesis, either by the secretion of toxic molecules to host cells, or by competing with bacterial rivals towards the colonization of a specific niche [1], [2]. Several studies have demonstrated the implications of T6SS in the virulence of *Vibrio cholerae* towards amoeba or animal/human host cells, of *Edwardsiella tarda* towards fishes, or of *Burkholderia* sp. towards animal host cells [5], [6], [24], [35], [36]. Similarly, high levels of antibodies directed against the HSI-1 Hcp protein of *Pseudomonas aeruginosa* have been detected in the sputum of cystic fibrosis patients [9]. With the observation that the HSI-1 T6SS is dedicated to the competition against other bacteria, including pathogens [8], [37], the presence of Hcp antibodies suggests that an intense bacterial warfare occurs in specific niches of animal or human bodies. As an inroad to better understand how T6SS are built, we initiated a structural, biophysical and functional characterization of the T6SS core components. We reported here the crystal structure of the TssJ lipoprotein, a critical core-component of Type VI secretion systems, and its interaction with TssM.

### TssJ structure and interaction with TssM

The TssJ-TssM complex is part of a larger complex involving the inner membrane TssL protein and the peptidoglycan-associated TagL protein [22]. This membrane sub-complex of T6SS therefore links both membranes and the peptidoglycan layer, forming a trans-envelope, periplasm spanning, structure. Few structures have been reported so far in T6SS: the Hcp and VgrG proteins which are structural/exported components that share homologies with bacteriophage subunits [9], [11]. This study thus reports the first structure of a T6SS membrane complex component and provides clues on specific interaction sites.

TssJ is a β-sandwich resembling transthyretin with two additional elements: a protruding loop and a small helical domain. Sequence alignment of the T6SS TssJ homologues showed that all TssJ proteins share these additional elements (Figure S4), suggesting that the EAEC TssJ structure likely constitutes a prototype for T6SS-associated TssJ proteins. Because these regions are not required for the canonical fold but are conserved among TssJ homologues, we thought they may represent potential site of functionality. Indeed, using *in vivo* assays, we found that deletion of the extended loop L1-2 abrogates Hcp secretion, while the helical domain is necessary for TssJ stability. *In vivo* and *in vitro* protein-protein interaction studies further showed that the L1-2 loop of TssJ is required for efficient interaction with the periplasmic domain of TssM. Although present in all TssJ homologues, this loop is not well conserved in terms of amino-acid composition and length. The sequence alignment presented Figure S4 reveals two TssJ sub-families based on the L1-2 length (5- or 8-amino-acids). Because this loop is a critical determinant of the TssJ-TssM interaction, one may hypothesize that these variations may modulate specificity between TssJ and TssM homologues. Specificity between these two proteins can therefore be a determinant during T6SS assembly to ensure proper recognition between the cognate subunits when several T6SS gene clusters are encoded within the same genome. SPR experiments showed that the K_d_ between TssJ and TssM was ∼2-4 ìM, while a 1∶1 stoichiometry was determined by MALS/QELS/UV/RI. Sub-domain dissection of TssM further demonstrated that TssJ does not interact with the TssM-ekto-Nt domain, but rather with the TssM-ekto-Ct domain.

### Organization of the T6SS outer shell

**Figure 5:**
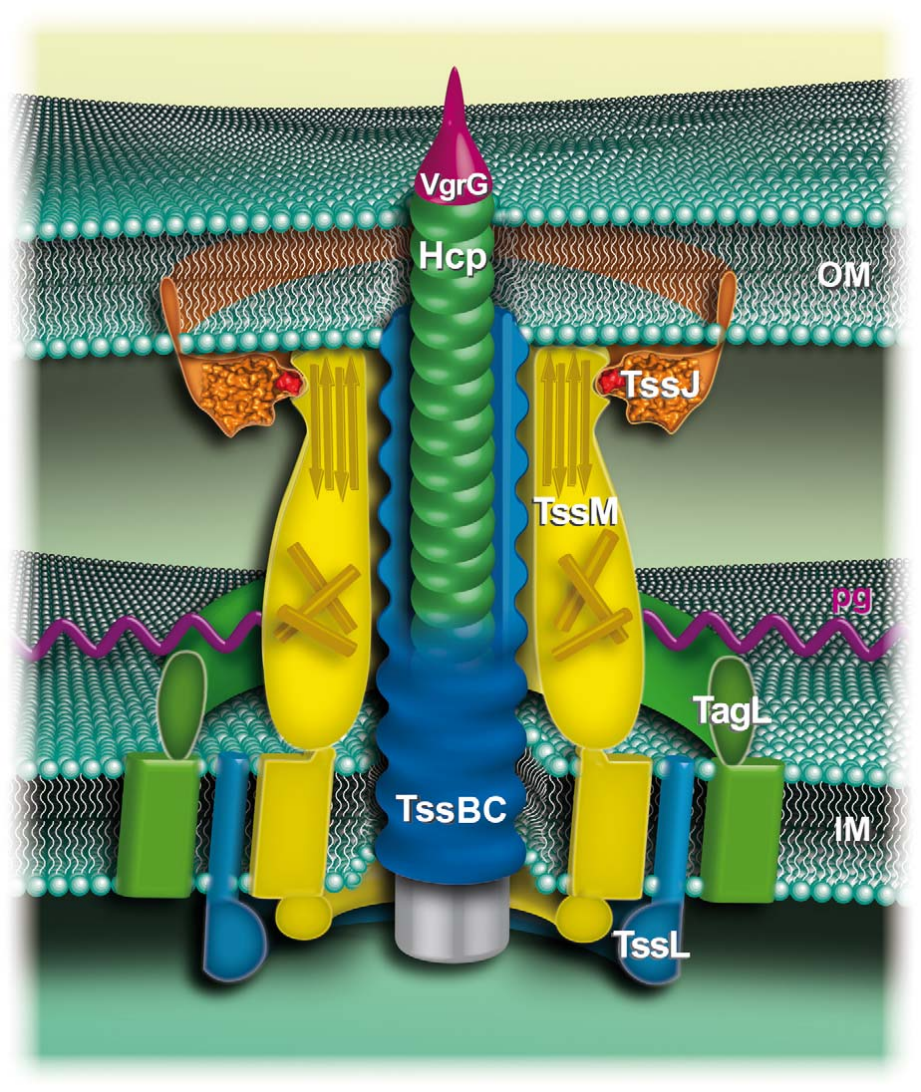
Schematic representation of the enteroaggregative *E. coli* T6SS. The outer (OM) and inner membranes (IM) are represented in light green. The T4 phage-like central puncturing device includes Hcp (green disks) and VgrG (purple). The “tail sheath” TssBC (VipAB) proteins are shown in blue, around the central Hcp/VgrG pilum. The TssBC proteins constituting a sheath encompassing the Hcp tube has not been evidenced but is speculated based on the similarities between the T6SS TssBC subunits and the bacteriophage T4 sheath [4], [17]. The three-transmembrane inner membrane TssM protein (yellow) interacts with the TssL IM protein (blue) [20]. TssL interacts with TagL (green), an IM protein that anchors the T6SS to the cell wall [22]. TssM C-terminal domain interacts with a loop of the outer membrane lipoprotein TssJ [this study]. In this model, the TssL-TagL-TssM-TssJ complex forms a trans-envelope spanning channel.

TssM-ekto-Ct domain localization in the sequence, opposite to the N-terminal trans-membrane helices imbedded in the IM, suggests that it should be close to the outer membrane. The hypothesis of the vicinity of TssM-ekto-Ct with the OM is further reinforced by the interaction of TssM-ekto-Ct with TssJ. However, formation of a T6SS outer shell based on TssM implies the establishment of lateral TssM-TssM interactions. The monomeric state of TssM-ekto in solution, as determined by MALS/QELS/UV/RI experiments, as well as the SPR data, are not in favor of this hypothesis. However, these interactions may still be established with the full length TssM molecules, since they could interact through their transmembrane helices. Our data, as well as the current knowledge on T6SS, made it possible to suggest a T6SS topology model (see Figure 5). As previously reported, TssL, TagL and TssM interact at the IM [22] whereas our data showed that the C-terminal domain of TssM interacts with TssJ at the OM (Figure 5). We propose that the TssL-TagL-TssM-TssJ complex may therefore form a trans-envelope spanning channel, as exemplified in other secretion systems [38]-[41].

### A chaperone role of TssJ?

The TssJ core domain (residues 2-155) is anchored to the membrane through a thioether linkage between Cys1 and a diacylglycerol. Residues 2-22 have been found to be non-ordered in the crystal structure, providing to TssJ a wide radius of periplasm exploration for catching TssM. Interestingly, the TssM-ekto-Ct domain is predicted to be essentially formed by β-strands. One interesting hypothesis that remains to be tested is that the TssJ lipoprotein may contribute to the folding or the stability of the TssM-ekto-Ct domain. A chaperone-like role for lipoproteins has already been demonstrated in most bacterial secretion systems: in Type II secretion systems (T2SS), the GspS lipoprotein acts as a pilotine to allow the passage of the GspD secretin through the periplasm and help to its proper insertion in the outer membrane [42]. Similarly, in T3SS, cognate lipoproteins serve as insertion helper for outer membrane secretins [43], [44]. More generally, lipoproteins associated with cell envelope spanning structures are often involved in machine assembly or nucleation factor. In T4SS, the VirB7 lipoprotein is covalently linked to the VirB9 outer-membrane associated component through a disulfide bridge [45], is required for stability of VirB9 and of the VirB9-VirB10 outer membrane translocon [39], and has been suggested to be necessary for the early stages of T4SS biogenesis [46]–[48]. Interestingly, the TssJ fold is similar to that of the *P. aeruginosa* T3SS-associated ExsB outer membrane lipoprotein [32]. This observation suggests that these two structures may represent a family of lipoproteins associated with macromolecular systems sharing a common evolutionary history. It is noteworthy that ExsB does not exhibit the L1-2 loop and the helical domain found in TssJ (Figure S3) highlighting the specific role of these two elements in Type VI secretion.

### Drugability of the TssM-TssJ interaction

Our data showed that the 4 amino-acids protruding loop L1-2 of TssJ is required for a functional TssJ-TssM interaction. This should be analyzed in light of the SPR experiments, reporting a K_d_ value in the µM range, suggesting that the interaction does not involve a large surface area of interaction, such as the 600-900 Å^2^ typical of Fab/protein interfaces that would assemble with K_d_ values down to the nM range [49]. This observation as well as the complete loss of TssJ-TssM interaction when the L1-2 loop is deleted let us to conclude that there is no other interaction area between TssM and TssJ. Interestingly, the size of the L1-2 loop is comparable with that of medium sized organic molecules that could therefore be good candidates to fit into the TssM complementary cavity and compete for TssJ binding. T6SS being important determinant of pathogenesis, biofilm formation or inter-bacterial competition, this observation paves the way for the identification of anti-microbial molecules targeting T6SS assembly [50].

## Materials and Methods

### Bacterial strains, medium, growth conditions and chemicals

*Escherichia coli* K12 DH5α was used for cloning procedures. The enteroaggregative *E. coli* strain 17-2 (kindly provided by Arlette Darfeuille-Michaud, University of Clermont-Ferrand, France) and its Δ*tssJ* derivative [Δ*sciN*; 21] were used for this study. EAEC strains were routinely grown in LB medium at 37°C with shaking. Plasmids were maintained by the addition of ampicillin (200 µg/ml) or kanamycin (100 µg/ml).

### Constructions for *in vivo* studies

Constructions of plasmid pSciN_HA_ (encoding the WT EAEC TssJ protein fused to a C-terminal HA epitope) and pHcp_FLAG_ (encoding the Hcp protein fused to a C-terminal FLAG epitope) have been previously reported [21]. Deletion of the L1-2 loop has been introduced into pSciN_HA_ by site-directed mutagenesis using mutagenic primers annealing upstream and downstream of the sequence to be deleted (5′-CCAGGGAGGCCATTAACACCGGTGGCGCCTCGGTTGTGGTGCGGATTTATC and 5′-GATAAATCCGCACCACAACCGAGGCGCCACCGGTGTTAATGGCCTCCCTGG). pSciSp, encoding the EAEC TssM-ekto domain (amino-acids 386 to 1129) fused to a signal peptide for periplasm targeting, has been constructed by a double PCR technique using pASK-IBA4 as template and oligonucleotides IBA-Sp5 (5′-CGCTACCGTAGCGCAGGCCGCTAGCGATTATAAAGACGACGATGACAAAAGTCTGGTTGCTGAAGTACAGGAACAGATTCGTCCG [sequence encoding the FLAG epitope tag underlined]) and IBA-Sp3 5′-GCCTTTTTCGAACTGCGGGTGGCTCCATCAGTCAGTCTCCTCCACGGTATCCCCGG). Plasmid encoding TssM-ekto-Nt (amino-acids 386 to 973) has been obtained by site-directed mutagenesis by introduction of a stop codon at codon Asn974 using pSciSp as template and oligonucleotides 5′- GGATGTGGCGTTCACCACAGGTTAAGCGGGGCTGCATTTTGAGCTGC and 5′- GCAGCTCAAAATGCAGCCCCGCTTAACCTGTGGTGAACGCCACATCC. Plasmid encoding TssM-ekto-Ct (amino-acids 972 to 1129) has been constructed by a double PCR technique using pASK-IBA4 as template and oligonucleotides IBA-Spβ5 (5′-CGCTACCGTAGCGCAGGCCGCTAGCGATTATAAAGACGACGATGACAAAGGTAACGCGGGGCTGCATTTTGAGCTGCG) and IBA-Sp3. Primers were obtained from custom oligonucleotides synthesized by Eurogentec. Polymerase Chain Reactions (PCR) were performed with a Biometra thermocycler, using the Pfu Turbo DNA polymerase (Stratagene; La Jolla, CA). All constructs have been verified by DNA sequencing (GATC).

### *In vivo* Hcp release assay

Supernatant and cell fractions have been separated as previously described [21]. Briefly, 2×10^9^ cells producing FLAG epitope-tagged Hcp were harvested and collected by centrifugation at 2,000 × *g* for 5 min. The supernatant fraction was then subjected to a second low-speed centrifugation and then at 16,000 × *g* for 15 min. The supernatant was then filtered on sterile polyester membranes with a pore size of 0.2 µm (membrex 25 PET, membraPure GmbH) before precipitation with trichloroacetic acid (TCA) 15%. Cells and precipitated supernatant were then resuspended in loading buffer and analyzed by SDS-PAGE and immunoblotting with the anti-FLAG M2 monoclonal antibody (Sigma-Aldrich). As control for cell lysis, Western blots were probed with antibodies raised against the periplasmic TolB protein.

### *In vivo* co-immunoprecipitation assays

Co-immunoprecipitation experiments were performed essentially as previously described [51]. 2×10^9^ exponentially growing cells were harvested, washed with 20 ml of 10 mM sodium phosphate buffer (NaPi, pH 6.8), and resuspended in NaPi buffer supplemented with *para-*formaldehyde 1%. After incubation at room temperature for 20 minutes, the cross-linking reaction was quenched by the addition of 0.3 M Tris-HCl pH 6.8, and the cells were washed twice in Tris-HCl 20 mM pH 6.8. The cell pellet was then subjected to solubilization for 30 min at 37°C in TES (Tris-HCl 10 mM, pH 7.5, EDTA 5 mM, SDS 1%) in presence of protease inhibitors (Complete, Roche), and diluted 15-fold in TNE (Tris-HCl 10 mM, pH 7.5, EDTA 5 mM, NaCl 150 mM) supplemented with 1% Triton X-100. After incubation 2 hours at room temperature with vigorous shaking, the extract was centrifuged 15 min at 18000 x *g* to remove unsolubilized material. Supernatants were then incubated overnight at 4°C with anti-HA antibody coupled to Agarose-Protein G beads (Roche). Beads were then washed twice with TNE supplemented with 1% Triton X-100, once in TNE supplemented with 0.1% Triton X-100 and Tween 0.1% and once in TNE supplemented with 0.1% Triton X-100. The immunoprecipitated material was heated in loading buffer prior to analyses by SDS-PAGE and immunoblotting.

### Constructions for *in vitro* studies and purification procedures

*tssJ* and *tssM-ekto* of enteroaggregative *Escherichia coli* strain 17-2 were cloned into pETG-20A (a kind gift from Dr Arie Geerlof, EMBL, Hamburg) expression vector according to standard Gateway™ protocols. The final constructs encoded the target genes and an N-terminal fusion with hexahistidine tagged thioredoxine followed by a TEV protease cleavage site. Both plasmids were transformed in *Escherichia coli* T7 Iq pLysS (New England Biolabs) expression strain. Cells were grown at 37°C in Terrific Broth until the OD_600_ reached 0.9 and the *tssJ* or *tssM* expression was inducted with 0.5 mM isopropyl-β-thio-galactoside (IPTG) overnight at 25°C and 17°C, respectively. After cells harvesting, the lysis was done by adding 0.25 mg/ml lysozyme, followed by sonication. Soluble protein was separated from inclusion bodies and cell debris by 30 min of centrifugation at 20,000 g. We used an AKTA FPLC system to four steps of purification: a Ni^2+^ affinity chromatography (HisTrap 5 ml GE Healthcare) with a step gradient of 250 mM Imidazole, an overnight TEV His protease digestion at 4°C with a 1∶10 (w/w) protease: protein ratio, a second Ni^2+^ affinity chromatography; a preparative Superdex 75 (GE Healthcare) gel filtration run in 20 mM Tris pH 8, 100 mM NaCl. Gel filtration of TssM-ekto was performed in a preparative Superose 6 in 20 mM Tris pH 8.0, 200 mM NaCl, 5% glycerol.

### *In vitro* co-purification assays

Co-purification of TssM-ekto and TssJ was performed in three steps on a AKTA FPLC system: a Ni^2+^ affinity chromatography in a step gradient of 250 mM Imidazole of the mixture Trx-(His6)-TssM-ekto and TssJ, an overnight TEV His protease digestion at 4°C with a 1∶10 (w/w) protease: protein ratio and a preparative Superose S6 (GE Healthcare) gel filtration run in 20 mM Tris pH 8.0, 200 mM NaCl, 5% glycerol.

### Biophysical experiments

Size exclusion chromatography (SEC) was performed on an Alliance 2695 HPLC system (Waters) using a KW803 and KW804 columns (Shodex) run in 10 mM HEPES pH 7.5, 150 mM NaCl at 0.5 ml/min. MALS, UV spectrophotometry, QUELS and RI were achieved with MiniDawn Treos (Wyatt Technology), a Photo Diode Array 2996 (Waters), a DynaPro (Wyatt Technology) and an Optilab rEX (Wyatt Technology), respectively, as described [26]. Mass and hydrodynamic radius calculation was done with ASTRA software (Wyatt Technology) using a *dn/dc* value of 0.185 mL/g.

Circular dichroism spectra of TssJ, TssJ-ΔL1-2 mutant and Trx-TssM-ekto were recorded in 20 mM NaH_2_PO_4_ pH 7,2, 150 mM NaCl using a Jasco J-810 spectropolarimeter.

The Surface Plasmon Resonance measurements were performed in a 10 mM Tris pH 8, 150 mM NaCl, 0.005% detergent TWEEN using a BIACORE X100 (BIAcore). The chip CM5 (BIAcore) was coated with TssJ N-terminal thioredoxine fusion with 600 response units (RU). We also used the inverse set-up, coating the chip CM5 with TssM-ekto N-terminal thioredoxine fusion with 3000 response units. Binding assays with TssJ N-terminal thioredoxine fusion covalently linked to the chip CM5 were performed with TssM N-terminal thioredoxine fusion at 10, 5, 2.5, 1.25, 0.625, 0.312 µM. We also performed the binding assays with the chip CM5 coated with TssM N-terminal thioredoxine fusion at 40, 20, 10, 5, 2.5, 1.25, 0.625, 0.312 µM. The signal from the uncoated reference cell and the buffer response was subtracted from all measurements. The K_D_ values were obtained using the fitting tool of the BIAevaluation software (BIAcore). A 1∶1 binding model was assumed in all cases.

### Crystallization and structure determination

TssJ crystallization trials were carried out in sitting-drop vapor diffusion method at 20°C in 96-well Greiner crystallization plates using a nanodrop-dispensing robot (Cartesian Inc.) [27]. Crystals grew in a few days by mixing 300 nL protein at 8mg/mL with 100 nL 2.2 M AmSO_4_, 0.2 M Na^+^-thiocyanate pH 6.0. Crystals were cryo-protected with mother-liquor supplemented with 20% ethylene glycol and flash frozen in liquid nitrogen. Some crystals were soaked in the cryo-protecting solution that also contained 0.5 M NaI and 0.5 M CsI. Two data sets were collected: a native data set (λ = 0.98011) and I-SAD (λ = 1.37760) at Proxima I beamline (SOLEIL, Gif-sur-Yvette, France) using an ADSC Q315r detector. Data processing and scaling were done using XDS, XSCALE [52], and POINTELESS (Table S3.). The crystals of TssJ belong to space group P3_1_21 with unit-cell parameters a = b = 78.07 Å, c = 46.95 Å. TssJ structure was solved by SIRAS with Sharp [53] and initial automatic building was performed with Buccaneer [54]. Manual model building was performed with COOT [55]. Refinement was carried out at 1.35 Å using Buster-TNT [56] and anisotropic refinement with REFMAC [57]. Structure analyzes was assisted by the PISA server [28] and electrostatic potential calculation was done with APBS [58].

#### Accession codes

The atomic coordinates and structure factors have been deposited at the Protein Data Bank with accession code 3RX9.

## Supporting Information

Figure S1 **EAEC TssJ analyzed by MALS/QELS/UV/RI experiments.** The protein mass was measured at 17260 ± 800 Da, a value close to that of the theoretical mass of 16,899 Da (Table S1).
(TIF)

Figure S2 **Stereo view of the Fo-Fc electron density map of TssJ depicted at 1 sigma level around Tyr 65.**
(TIF)

Figure S3 **Superimposition of the EAEC T6SS TssJ lipoprotein with the** ***P. aeruginosa*** **T3SS ExsB lipoprotein (PDB 2yjl** [**32**] **).** TssJ and ExsB exhibit a common transthyretin fold, but TssJ possesses an extra α-domain and an extended loop between strands 1 and 2 (indicated by the arrows). Panels A and B are rotated 90° from each other.
(TIF)

Figure S4
**Sequence alignment of enteroaggregative** ***E. coli*** **TssJ (gi218696620) with the 49 first hits obtained by blasting the NR database.** The secondary structures are depicted according to the TssJ crystal structure. The red arrow indicates the position at which the electron density starts in the X-ray structure. Note that the 33 first sequences do not present any insertion/deletions compared to the EAEC TssJ protein. The conserved residues are boxed in red. Other semi-conserved residues are depicted in red and boxed in blue. Sequence alignment has been performed with Multalin (http://multalin.toulouse.inra.fr/multalin/) [59] and ESPript [60]. (gi161504599, *Salmonella enterica* subsp. *arizonae*; gi288550304, *Enterobacter cancerogenus*; gi262041848, *Klebsiella pneumoniae* subsp. *rhinoscleromatis*; gi238895309, *K. pneumoniae*; gi 152970795 *K. pneumoniae* subsp. *pneumoniae* MGH 78578; gi206578921, *K. pneumoniae*; gi290508950, *Klebsiella* sp.; gi238792667, *Yersinia intermedia*; 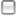
gi238783371, *Y. bercovieri*; 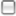
gi238798881, *Y. mollaretii*; 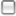
gi238790215, *Y. frederiksenii*; 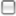
gi238795915, *Y. mollaretii*; 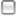
gi238751672, *Y. rohdei*; 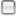
gi226328469, *Proteus penneri*; gi212712391, *Providencia alcalifaciens*; gi261347002, *Providencia rustigianii*; gi156932370, *Cronobacter sakazakii*; gi253991289, *Photorhabdus asymbiotica*; gi37528038, *Photorhabdus luminescens*; gi161504491, *Salmonella enterica* subsp. *arizonae*; gi292898109, *Erwinia amylovora* ATCC 49946; gi292489675, *Erwinia amylovora* CFBP1430; gi260597748, *Cronobacter turicensis*; gi156934209, *Cronobacter sakazakii*; gi206579068, *K. pneumoniae*; gi262039776, *K. pneumoniae* subsp. *rhinoscleromatis*; gi194434338, *Shigella dysenteriae*; gi26249236, *E. coli* CFT073; gi194426146, *E. coli* B171; gi307554802, *E. coli* ABU 83972; gi227888372, *E. coli* 83972; gi317491536, *Enterobacteriaceae bacterium*; gi149366543, *Y. pestis* CA88-4125; gi22126561, *Y. pestis* KIM10; 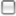
gi153947257, *Y. pseudotuberculosis* IP31758; gi270486848, *Y. pestis* KIMD27; gi170024810, *Y. pseudotuberculosis* YPIII; gi51595841, *Y. pseudotuberculosis* IP32953; gi108806780, *Y. pestis* Antiqua; gi153950286, *Y. pseudotuberculosis* IP31758; gi300715009, *Erwinia billingiae*; gi157370044, *Serratia proteamaculans*; gi296102178, *Enterobacter cloacae*; gi304398550, *Pantoea* sp.; gi290509839, *Klebsiella* sp.; gi238894433, *K. pneumoniae*; gi288935813, *K. variicola*; gi317049653, *Pantoea* sp.).(TIF)

Figure S5 **Representation of TssJ conserved residues.** TssJ is shown in ribbon representation and rainbow coloring from blue (N-terminus) to red (C-terminus); the sequence is represented above. Figure made with Pymol [61].
(TIF)

Figure S6 **Surface representation of TssJ.** **(A)** TssJ B-factors. The surface is colored according to B-factors values, from blue (low B-factors) to red (high-B-factors). **(B)** TssJ electrostatic potential. The surface is colored according to the contact electrostatic potential calculated with Pymol [61]. Positively charged areas are shown in blue and negatively charged areas are in red.
(JPG)

Figure S7 **Circular dichroism spectra of TssJ and TssM. (A)** CD spectra of TssM-ekto (black line) and TssM-ekto-Nt (blue line). **(B)** Comparison of the Wild-type TssJ (TssJ wt; black line) compared to the loop depleted mutant (TssJ-ΔL1-2; red line).
(TIF)

Table S1 **Biochemical characteristics of the proteins and domains reported in this work.**
(DOC)

Table S2 **Biophysical characteristics of the proteins and domains reported in this work.**
(DOC)

Table S3 **Data collection (PROXIMA 1 at SOLEIL) and refinement statistics.**
(DOC)
