# Supplementary figures and images for: Towards a Structural Comprehension of Bacterial Type VI Secretion Systems: Characterization of the TssJ-TssM Complex of an Escherichia coli Pathovar
Source: PLoS Pathog. 2011 Nov 10;7(11):e1002386. doi: 10.1371/journal.ppat.1002386 (PMC3213119; doi:10.1371/journal.ppat.1002386)

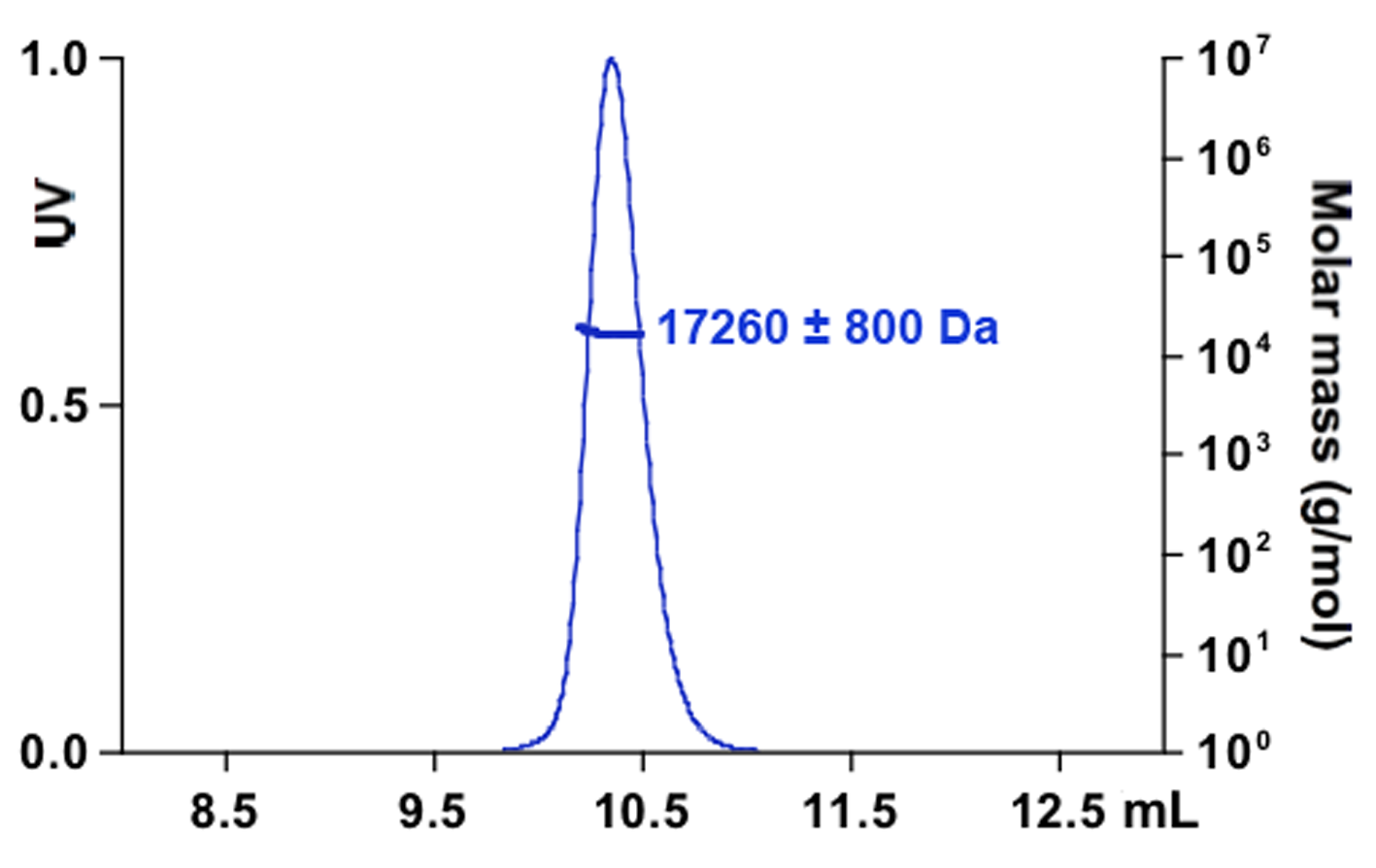

Supplement: Figure S1 — EAEC TssJ analyzed by MALS/QELS/UV/RI experiments. The protein mass was measured at 17260 ± 800 Da, a value close to that of the theoretical mass of 16,899 Da (Table S1). (TIF) [file ppat.1002386.s001.tif]

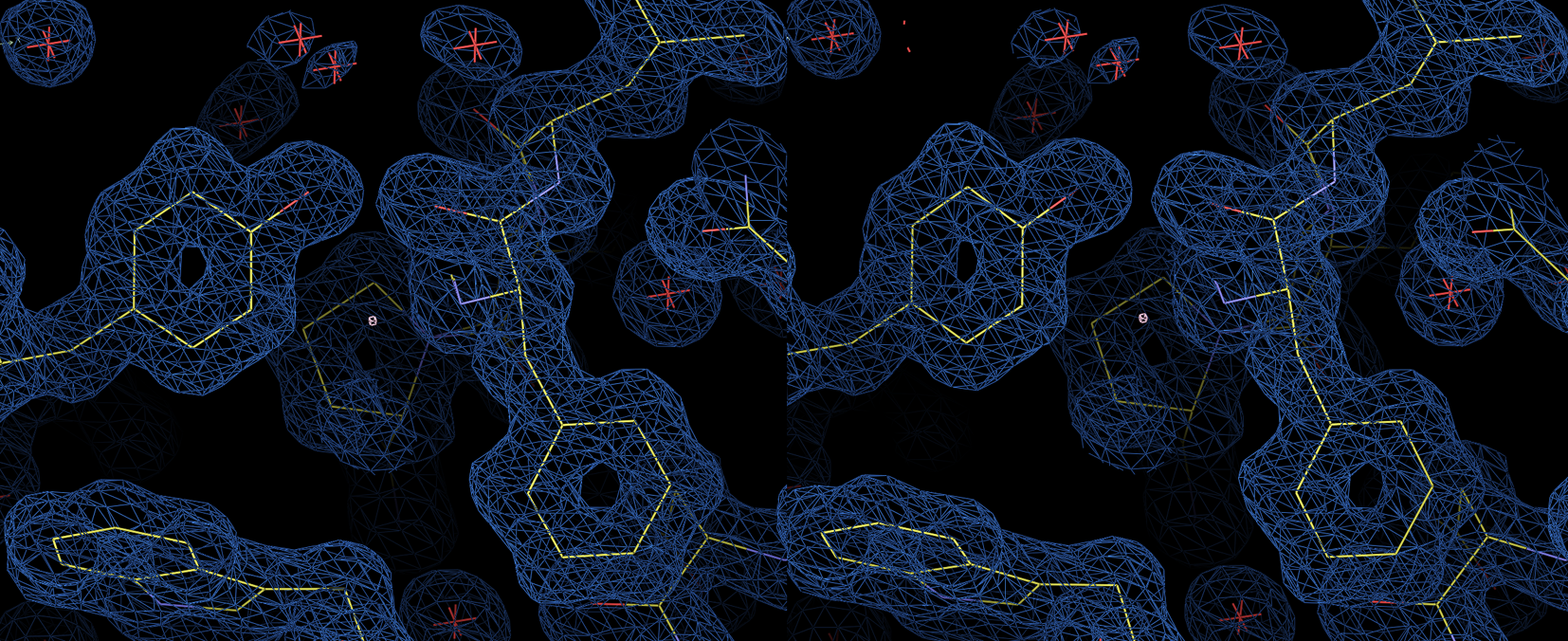

Supplement: Figure S2 — Stereo view of the Fo-Fc electron density map of TssJ depicted at 1 sigma level around Tyr 65. (TIF) [file ppat.1002386.s002.tif]

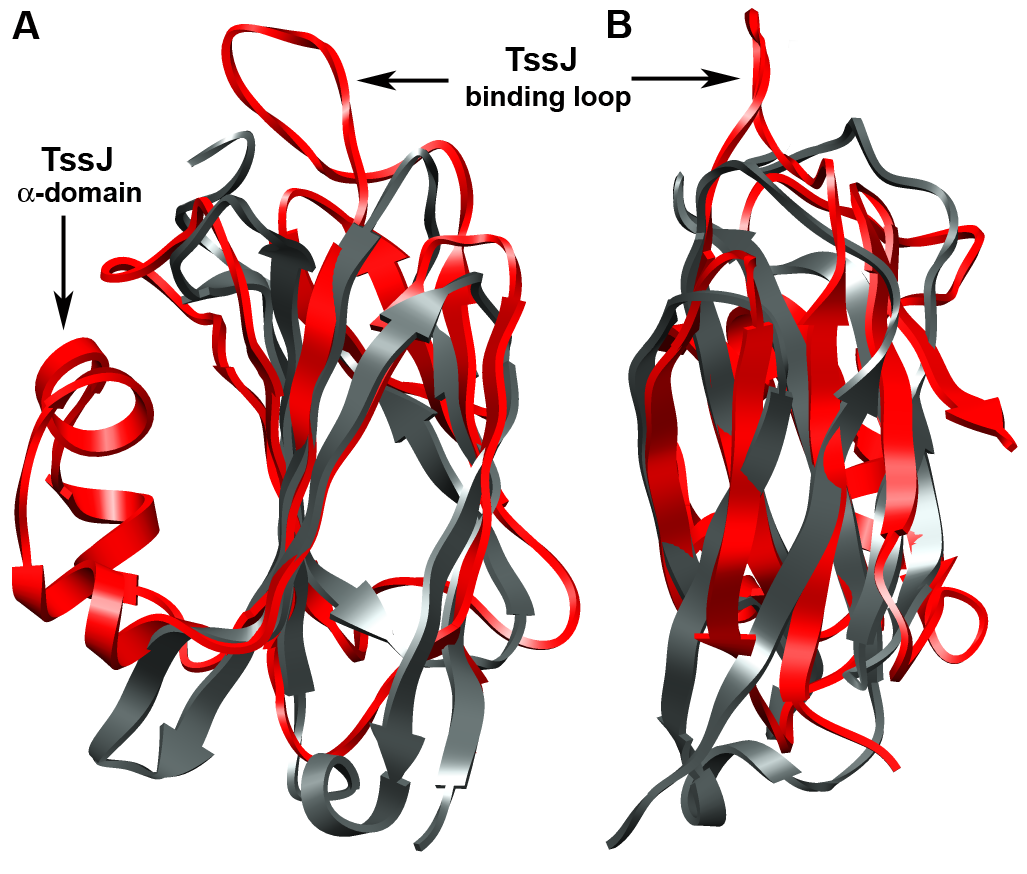

Supplement: Figure S3 — Superimposition of the EAEC T6SS TssJ lipoprotein with the P. aeruginosa T3SS ExsB lipoprotein (PDB 2yjl [32] ). TssJ and ExsB exhibit a common transthyretin fold, but TssJ possesses an extra α-domain and an extended loop between strands 1 and 2 (indicated by the arrows). Panels A and B are rotated 90° from each other. (TIF) [file ppat.1002386.s003.tif]

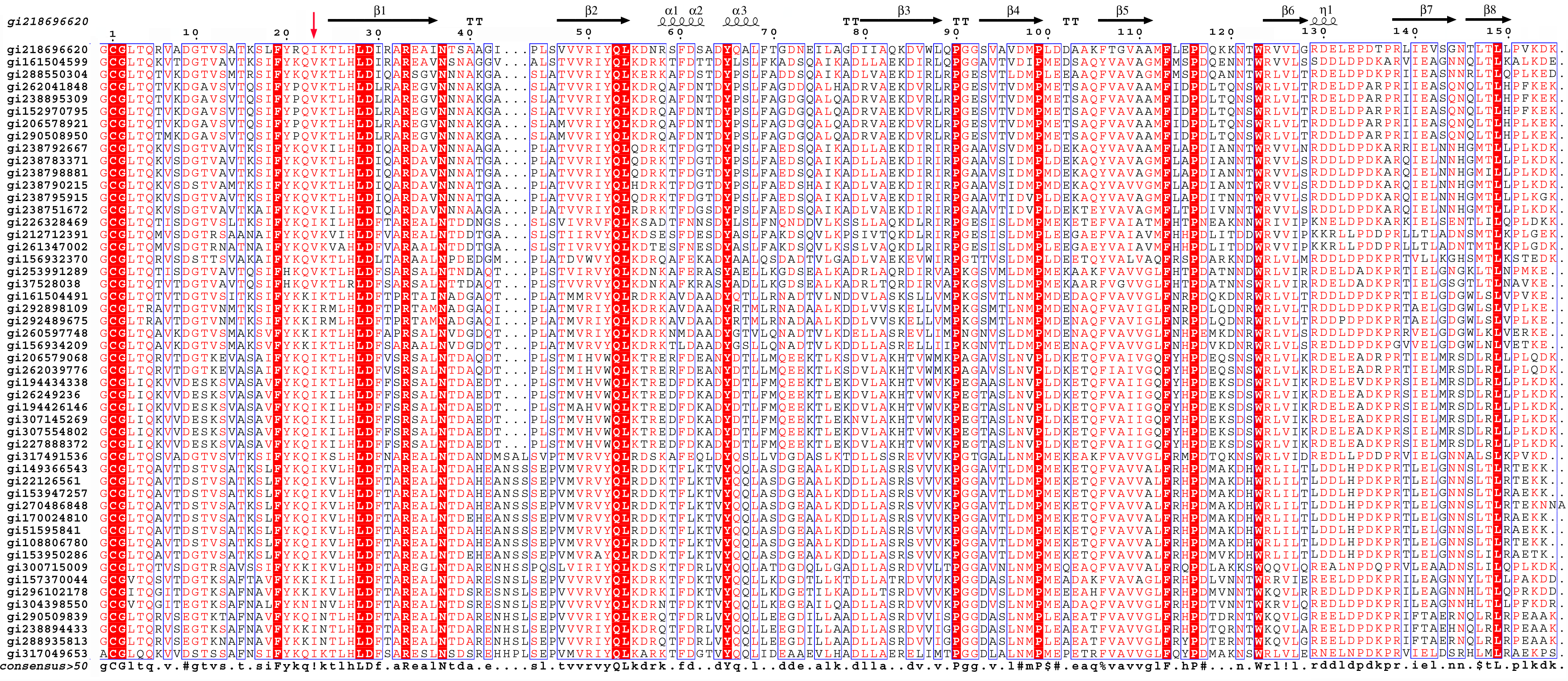

Supplement: Figure S4 — Sequence alignment of enteroaggregative E. coli TssJ (gi218696620) with the 49 first hits obtained by blasting the NR database. The secondary structures are depicted according to the TssJ crystal structure. The red arrow indicates the position at which the electron density starts in the X-ray structure. Note that the 33 first sequences do not present any insertion/deletions compared to the EAEC TssJ protein. The conserved residues are boxed in red. Other semi-conserved residues are depicted in red and boxed in blue. Sequence alignment has been performed with Multalin (http://multalin.toulouse.inra.fr/multalin/) [59] and ESPript [60]. (gi161504599, Salmonella enterica subsp. arizonae; gi288550304, Enterobacter cancerogenus; gi262041848, Klebsiella pneumoniae subsp. rhinoscleromatis; gi238895309, K. pneumoniae; gi 152970795 K. pneumoniae subsp. pneumoniae MGH 78578; gi206578921, K. pneumoniae; gi290508950, Klebsiella sp.; gi238792667, Yersinia intermedia; gi238783371, Y. bercovieri; gi238798881, Y. mollaretii; gi238790215, Y. frederiksenii; gi238795915, Y. mollaretii; gi238751672, Y. rohdei; gi226328469, Proteus penneri; gi212712391, Providencia alcalifaciens; gi261347002, Providencia rustigianii; gi156932370, Cronobacter sakazakii; gi253991289, Photorhabdus asymbiotica; gi37528038, Photorhabdus luminescens; gi161504491, Salmonella enterica subsp. arizonae; gi292898109, Erwinia amylovora ATCC 49946; gi292489675, Erwinia amylovora CFBP1430; gi260597748, Cronobacter turicensis; gi156934209, Cronobacter sakazakii; gi206579068, K. pneumoniae; gi262039776, K. pneumoniae subsp. rhinoscleromatis; gi194434338, Shigella dysenteriae; gi26249236, E. coli CFT073; gi194426146, E. coli B171; gi307554802, E. coli ABU 83972; gi227888372, E. coli 83972; gi317491536, Enterobacteriaceae bacterium; gi149366543, Y. pestis CA88-4125; gi22126561, Y. pestis KIM10; gi153947257, Y. pseudotuberculosis IP31758; gi270486848, Y. pestis KIMD27; gi170024810, Y. pseudotuberculosis YPIII; gi51595841, [file ppat.1002386.s004.tif]

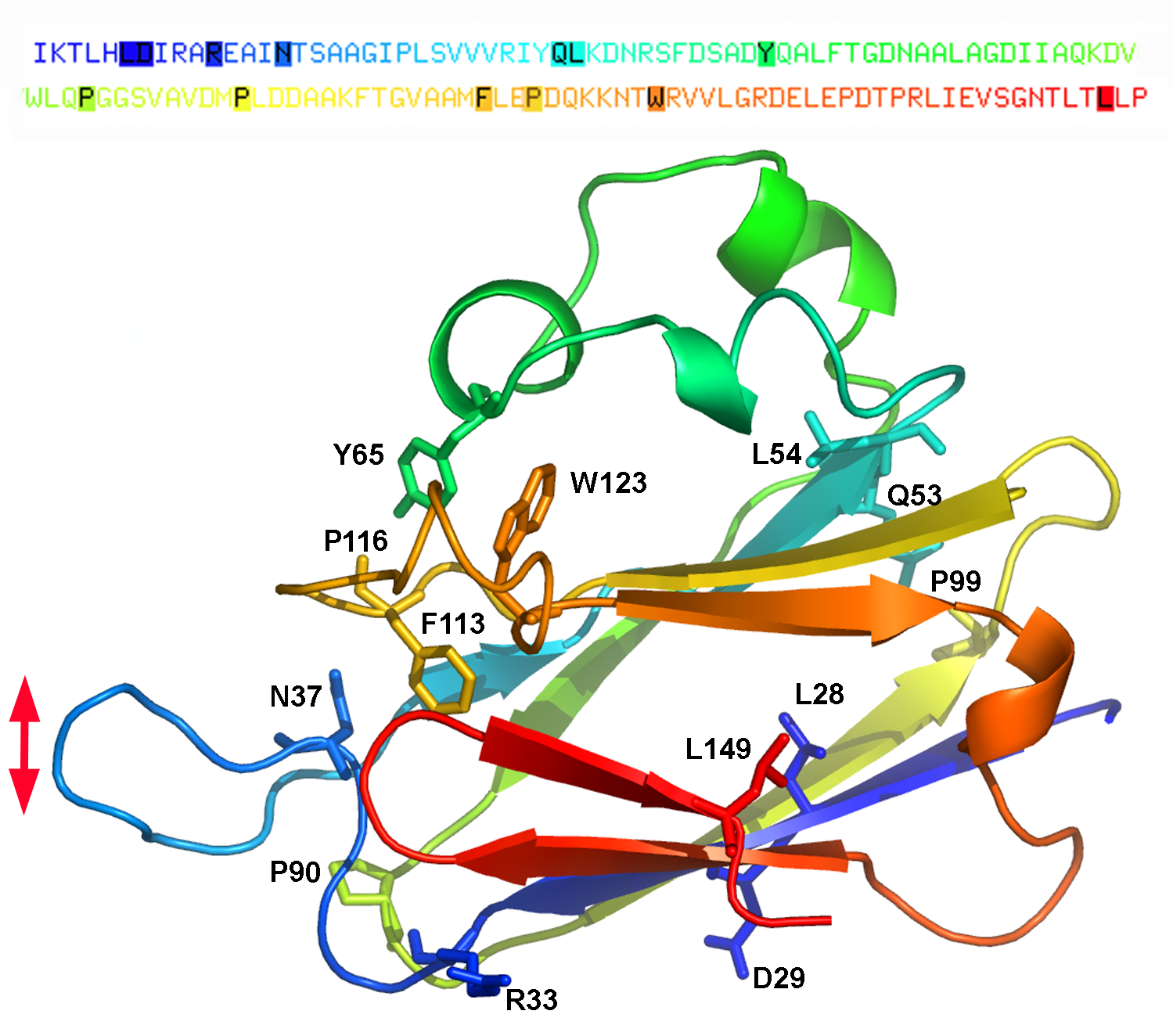

Supplement: Figure S5 — Representation of TssJ conserved residues. TssJ is shown in ribbon representation and rainbow coloring from blue (N-terminus) to red (C-terminus); the sequence is represented above. Figure made with Pymol [61]. (TIF) [file ppat.1002386.s005.tif]

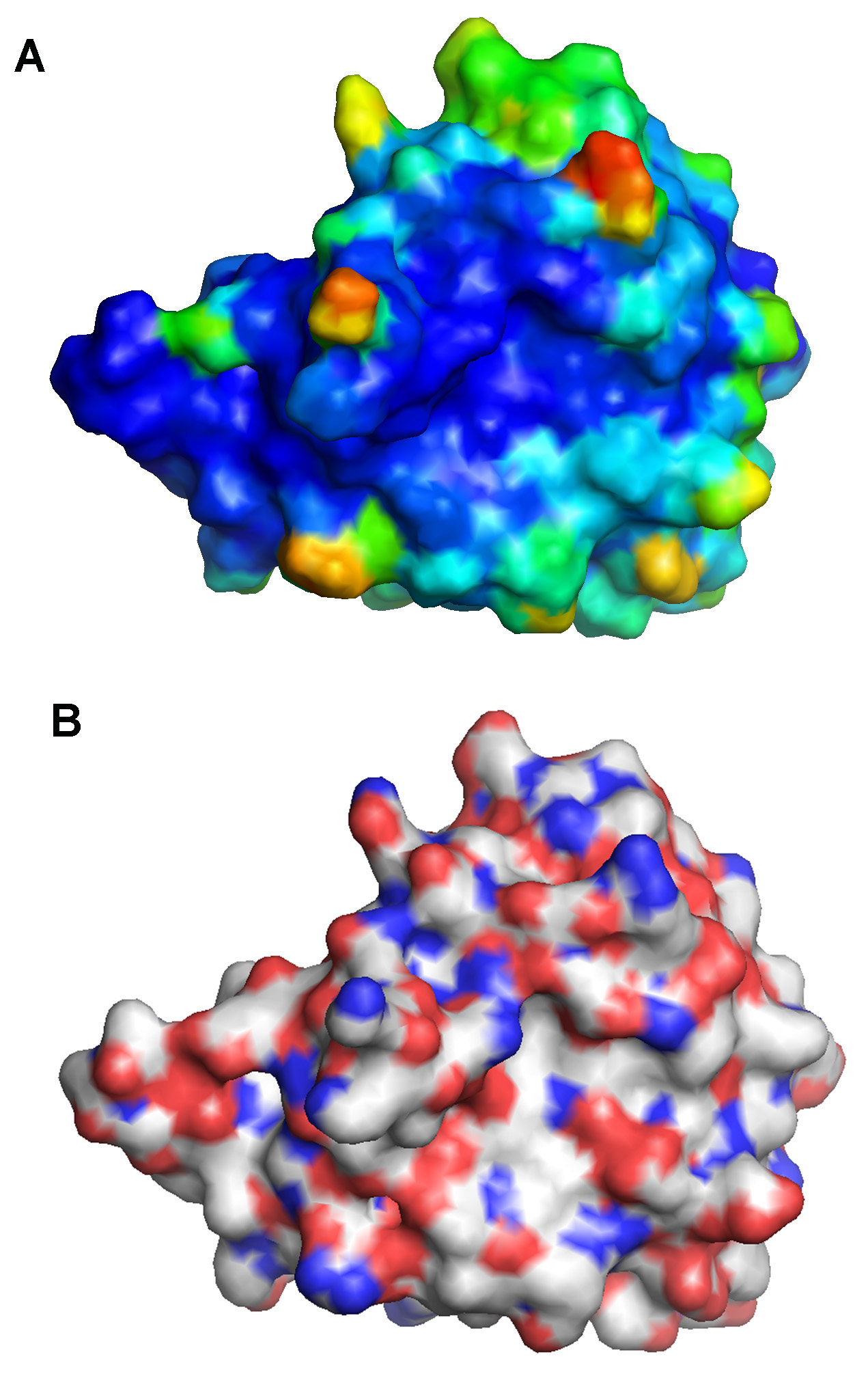

Supplement: Figure S6 — Surface representation of TssJ. (A) TssJ B-factors. The surface is colored according to B-factors values, from blue (low B-factors) to red (high-B-factors). (B) TssJ electrostatic potential. The surface is colored according to the contact electrostatic potential calculated with Pymol [61]. Positively charged areas are shown in blue and negatively charged areas are in red. (JPG) [file ppat.1002386.s006.jpg]

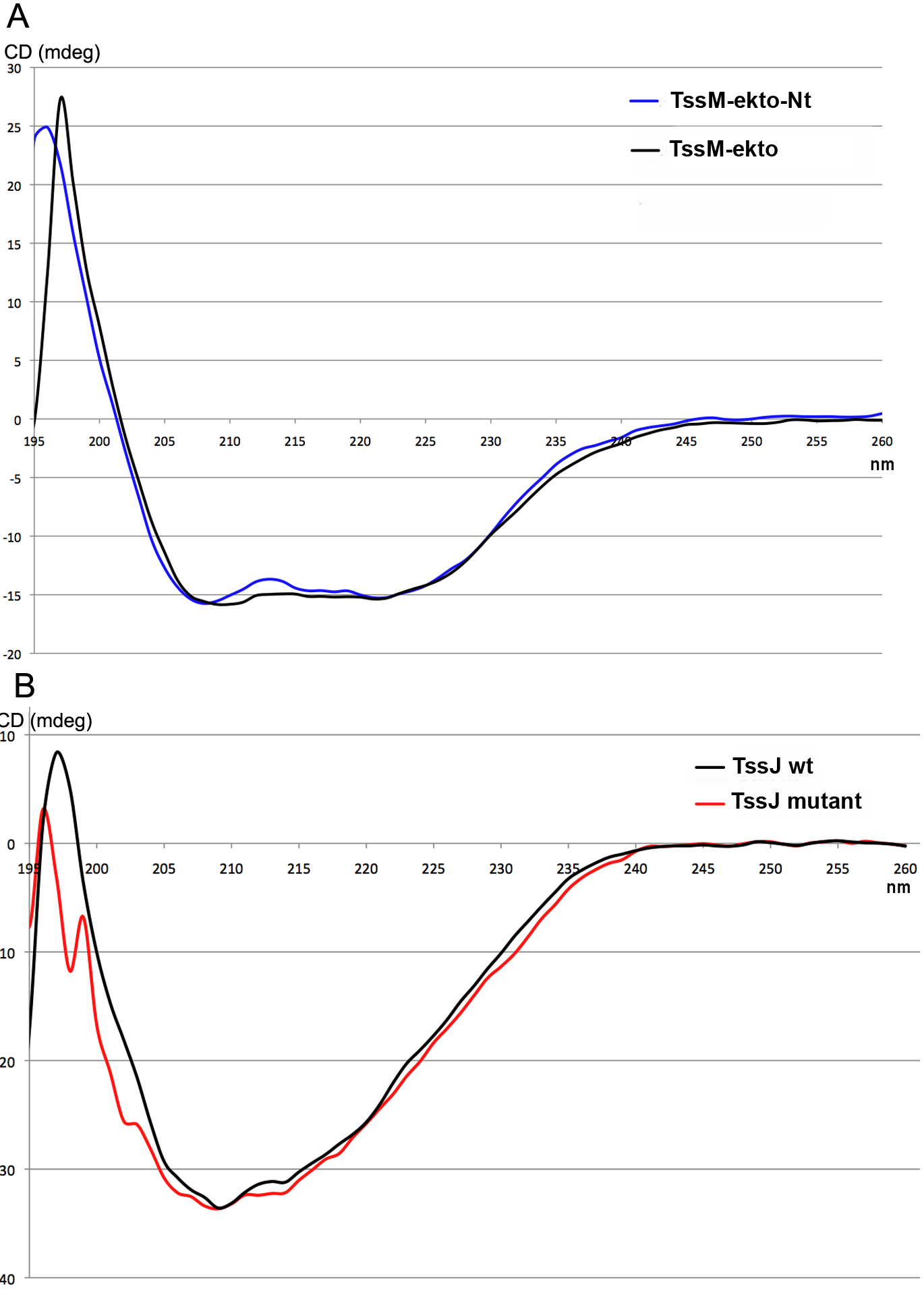

Supplement: Figure S7 — Circular dichroism spectra of TssJ and TssM. (A) CD spectra of TssM-ekto (black line) and TssM-ekto-Nt (blue line). (B) Comparison of the Wild-type TssJ (TssJ wt; black line) compared to the loop depleted mutant (TssJ-ΔL1-2; red line). (TIF) [file ppat.1002386.s007.tif]
