# Supplementary material for: Towards a Structural Comprehension of Bacterial Type VI Secretion Systems: Characterization of the TssJ-TssM Complex of an Escherichia coli Pathovar
Source: PLoS Pathog. 2011 Nov 10;7(11):e1002386. doi: 10.1371/journal.ppat.1002386 (PMC3213119; doi:10.1371/journal.ppat.1002386)
Supplement: Table S1 — Biochemical characteristics of the proteins and domains reported in this work. (DOC) [file ppat.1002386.s008.doc]

**Supporting Table S1**

**Table S1**. Biochemical characteristics of the proteins and domains reported in this work.

|  | Protein/domain | Nr res. | mass (kDa)^a^ | pI |
| --- | --- | --- | --- | --- |
| TssJ | 1-155 | 155 | 16,899 | 5.15 |
| TssJ-∆L1-2 | 1-38//43-155 | 151 | 16,474 | 5.15 |
| TssM | 1-1129 | 1129 | 126,979 | 9.04 |
| TssM ekto | 386-1129 | 744 | 83,070 | 6.19 |
| TssM ekto-Ct (beta) | 931-1129 | 198 | 21,785 | 5.10 |
| TssM ekto-Nt (alpha) | 386-930 | 545 | 61,303 | 7.13 |
| Trx-TssJ |  | 295 | 32,228 | 5.70 |

^a^ Theoretical mass.
