# Supplementary material for: Towards a Structural Comprehension of Bacterial Type VI Secretion Systems: Characterization of the TssJ-TssM Complex of an Escherichia coli Pathovar
Source: PLoS Pathog. 2011 Nov 10;7(11):e1002386. doi: 10.1371/journal.ppat.1002386 (PMC3213119; doi:10.1371/journal.ppat.1002386)
Supplement: Table S2 — Biophysical characteristics of the proteins and domains reported in this work. (DOC) [file ppat.1002386.s009.doc]

**Supporting Table S2.**

**Table S2**. Biophysical characteristics of the proteins and domains reported in this work.

|  | Stability/  Solublitiy (mg/ml) | DLS/Wyatt  (MW kDa) | Interaction with TssJ (GF/SDS) | Biacore  (k_on_/k_off_/  Kd) |
| --- | --- | --- | --- | --- |
| TssJ | 20+ | 17,260 |  |  |
| TssJ ΔL1-2 | 20+ | ND |  |  |
| TssM ekto-Ct (beta) | ND | ND | + | ND |
| TssM ekto-Nt (alpha) | 0.9 | ND | - | - |
| TssM ekto | 9 | 84,900 | + | 2-4 µM |
| TssM-TssJ | 20 | 99,970 |  |  |

ND, not determined.
