# Supplementary material for: Towards a Structural Comprehension of Bacterial Type VI Secretion Systems: Characterization of the TssJ-TssM Complex of an Escherichia coli Pathovar
Source: PLoS Pathog. 2011 Nov 10;7(11):e1002386. doi: 10.1371/journal.ppat.1002386 (PMC3213119; doi:10.1371/journal.ppat.1002386)
Supplement: Table S3 — Data collection (PROXIMA 1 at SOLEIL) and refinement statistics. (DOC) [file ppat.1002386.s010.doc]

**Supporting Table S3.**

**Table S3**. Data collection (PROXIMA 1 at SOLEIL) and refinement statistics.

|  | Native |
| --- | --- |
| **DATA COLLECTION** |  |
| Space group /cell | P3_1_2 1 / a=b=78.07 Å, c=46.98 Å |
| Wavelength (Å) | 0.98011 |
| Resolution limits^a^ (Å) | 70.00-1.35 (1.39-1.35) |
| Rmerge^a^ (%) | 5.3 (31.1) |
| Nr. of observations^a^ | 206066 (5319) |
| Nr. unique reflections^a^ | 35308 (2117) |
| Mean((I)/sd(I)) ^a^ | 18.1 (2.8) |
| Completeness^a^ (%) | 96.6 (79.5) |
| Multiplicity^a^ | 5.8 |
| **REFINEMENT** |  |
| Resolution^a^ (Å) | 67-1.35 (1.385-1.35) |
| Nr of reflections^a^ | 33535 (1938) |
| Nr of protein/water atoms | 1161/202 |
| Nr test set reflections | 1683 |
| Rwork/Rfree^a^ (%) | 14 /16.1 (20/23.7) |
| r.m.s.d.bonds(Å)/angles (°) | 0.011/ 1.58 |
| B-wilson / B-average | 13.4/16.9 |
| Coot’s ramachandran  Preferred / allowed % | 99.3 / 0.7 |

^a^ parenthesis refer to the highest resolution bin.
